# Ipsilateral lower extremity joint involvement increases the risk of poor pain and function outcomes after hip or knee arthroplasty

**DOI:** 10.1186/1741-7015-11-144

**Published:** 2013-06-05

**Authors:** Jasvinder A Singh, David G Lewallen

**Affiliations:** 1 Medicine Service and Center for Surgical Medical Acute care Research and Transitions (C-SMART), Birmingham VA Medical Center, Birmingham, AL, USA; 2 Department of Medicine at School of Medicine, and Division of Epidemiology at School of Public Health, University of Alabama, Birmingham, AL, USA; 3 Department of Orthopedic Surgery, Mayo Clinic College of Medicine, Rochester, MN, USA

**Keywords:** Arthroplasty, Ipsilateral, Joint replacement, Outcomes, Risk factors, Total hip replacement, Total knee replacement

## Abstract

**Background:**

Poor pain and function outcomes are undesirable after an elective surgery such as total hip or knee arthroplasty (THA/TKA). Recent studies have indicated that the presence of contralateral joint influences outcomes of THA/TKA, however the impact of ipsilateral knee/hip involvement on THA/TKA outcomes has not been explored. The objective of this study was to assess the association of ipsilateral knee/hip joint involvement on short-term and medium-term pain and function outcomes after THA/TKA.

**Methods:**

In this retrospective study of prospectively collected data, we used the data from the Mayo Clinic Total Joint Registry to assess the association of ipsilateral knee or hip joint involvement with moderate to severe pain and moderate to severe activity limitation at 2-year and 5-year follow-up after primary and revision THA and TKA using multivariable-adjusted logistic regression analyses.

**Results:**

At 2 years, 3,823 primary THA, 4,701 primary TKA, 1,218 revision THA and 725 revision TKA procedures were studied. After adjusting for multiple covariates, ipsilateral knee pain was significantly associated with outcomes after primary THA (all *P* values <0.01): (1) moderate to severe pain: at 2 years, odds ratio (OR), 2.3 (95% confidence interval (CI) 1.5 to 3.6); at 5 years, OR 1.8 (95% CI 1.1 to 2.7); (2) moderate to severe activity limitation: at 2 years, OR 3.1 (95% CI 2.3 to 4.3); at 5 years, OR 3.6 (95% CI 2.6 to 5.0). Ipsilateral hip pain was significantly associated with outcomes after primary TKA (all *P* values <0.01): (1) moderate to severe pain: at 2 years, OR 3.3 (95% CI 2.3 to 4.7); at 5 years, OR 1.8 (95% CI 1.1 to 2.7); (2) moderate to severe activity limitation: at 2 years, OR 3.6 (95% CI 2.6 to 4.9); at 5 years, OR 2.2 (95% CI 1.6 to 3.2). Similar associations were noted for revision THA and TKA patients.

**Conclusions:**

To the best of our knowledge, this is the first study showing that the presence of ipsilateral joint involvement after THA or TKA is strongly associated with poor pain and function outcomes. A potential way to improve outcomes is to address ipsilateral lower extremity joint involvement.

## Background

Total hip and total knee arthroplasty (THA and TKA) are successful surgical treatments for end stage arthritis, a frequent cause for disability and work limitations [1]. THA and TKA are associated with significant improvement in pain, function and quality of life [2]. The public health significance of joint arthroplasty is enormous, given that the combined annual volume of primary THA and TKA exceeded 1.1 million in 2009 in the USA [3]. The incidence of arthroplasty is increasing exponentially in the US and other countries [4-7]. However, more than 10% of the patients continue to have refractory pain and/or significant functional limitation even years after THA and TKA [8]. Identification of significant contributors to this residual pain and functional limitation may provide insights into potential strategies to improve outcomes, particularly where the risk factors are modifiable.

Recent studies have demonstrated an association of contralateral extremity strength [9] and contralateral knee pain [10] on outcomes 2 to 3 years after TKA. To the best of our knowledge, there are no published studies describing the effect of ipsilateral lower extremity joint involvement on intermediate-term patient-reported outcomes (PROs), that is, pain and function after hip or knee arthroplasty. We hypothesized that involvement of an ipsilateral knee in patients with THA and an ipsilateral hip in patients with TKA at post-arthroplasty follow-up would be associated with a higher risk of moderate to severe index arthroplasty pain and moderate to severe functional limitation.

## Methods

In this study, we used the data collected prospectively in the Mayo Total Joint Registry.

The Mayo Clinic Institutional Review Board approved this study and all investigations were conducted in conformity with ethical principles of research. The Mayo Total Joint Registry collects prospective data on all joint replacements performed at the Mayo Clinic, including patient demographics, date of last evaluation, surgical complications and pain and function assessments [11]. Validated Mayo Hip [12] and Mayo Knee [13] questionnaires containing pain and function questions have been administered to all patients and these data have been captured electronically starting in 1993. These questionnaires are mailed to patients, administered during clinic visits or by telephone by experienced, dedicated joint registry staff at 2-year and 5-year timepoints after THA or TKA. The pain and function questions analyzed in this study are same as those in the Harris Hip Score [14] and Knee Society Score [15], the most widely used questionnaires for post-THA and post-TKA assessment, respectively, that have face, content and construct validity. Study inclusion criteria were (1) patients had undergone a primary or revision THA or TKA between 1993 to 2005 at Mayo Clinic, Rochester, MN, USA; and (2) had responded to both pre-surgery Mayo Hip or Knee questionnaire and at least one post-surgery Mayo Hip or Knee questionnaire (either 2-year or 5-year follow-up survey).

### Predictor variable and its definition

The main predictor of interest was the presence of ipsilateral knee involvement in patients with THA and ipsilateral hip involvement in patients with TKA, assessed at 2-year and 5-year follow-up time-points, as part of self-reported knee and hip questionnaires. Ipsilateral hip/knee involvement was assessed by response to the following question: ‘Please indicate if your activities are limited by other joints (mark all that apply): none, right hip, left hip, right knee and left knee’. For patients undergoing THA, this meant involvement of the same-sided knee joint, and for patients undergoing TKA, same-sided hip involvement.

### Outcomes of interest

The following outcomes were assessed at 2 years and 5 years after THA and TKA.

(1) Moderate to severe hip pain (for THA): responses to a question ‘Do you have pain in the hip in which the joint was replaced?’, with responses of no pain, slight, moderate and severe. Moderate and severe pain categories were combined as per an *a priori* clinical decision, similar to our previous studies [16], since moderate to severe pain after THA is undesirable. This question is similar to the pain question in the Harris Hip Score [12].

(2) Moderate to severe knee pain (for TKA): responses to a question regarding pain in knee similar to the pain question in the Knee Society Scale [17], ‘Do you have pain in the knee in which the joint was replaced?’, with responses of no pain, mild (occasional), stairs only, walking and stairs (combined into reference category); moderate (occasional), moderate (continuous) and severe pain combined into moderate to severe pain, similar to previous studies [18].

(3) Moderate to severe activity limitation: for THA patients, responses to questions regarding limitations in seven activities including walking, stairs, putting on shoes/socks, picking up objects from the floor, sitting, getting in/out of the car and rising from a chair were categorized into ‘no’, ‘mild’, ‘moderate’ or ‘severe’ limitation for each activity. The presence of three or more activities with moderate or severe limitation was classified as overall moderate to severe activity limitation (reference, all other categories), as previously described [19]. For TKA patients, moderate to severe activity limitation was defined as presence of moderate or severe limitation in two or more of the three activities queried (walking, stairs, rising from chair), as previously described [18].

### Covariates of interest

These were selected based on documented (or suspected) association with arthroplasty outcomes and included: (a) patient characteristics: age and gender (unmodifiable) and body mass index (BMI) and comorbidity (modifiable); (b) American Society of Anesthesiologists (ASA) score; (c) operative diagnosis; (d) preoperative moderate to severe pain and function; (e) implant fixation; (f) psychological morbidity: depression and anxiety; and (g) health care access: distance from the medical center.

These variables were categorized as follows: (1) age: categorized as previously [19] into ≤60, 61 to 70, 71 to 80 and >80; (2) gender (female vs. male); (3) BMI: categorized based on World Health Organization (WHO) classification into ≤25, 25.1 to 29.9, 30 to 34.9, 35 to 39.9 and ≥40; (4) comorbidity: continuous variable (5-point increase) measured by Deyo-Charlson score, a validated comorbidity measure [20] and the most commonly used comorbidity measure consisting of a weighted scale of 17 comorbidities (including cardiac, pulmonary, renal, hepatic disease, diabetes, cancer, AIDS and so on), expressed as a summative score where a higher score indicates more comorbidity; (5) ASA physical status score: a validated measure of perioperative and postoperative outcomes categorized as class I to II vs. III to IV [21]; (6) operative diagnosis: categorized as osteoarthritis, inflammatory arthritis (including rheumatoid arthritis) or other for primary TKA; osteoarthritis, inflammatory arthritis (including rheumatoid arthritis), avascular necrosis or other for primary THA; loosening/wear/osteolysis, dislocation/fracture/instability/non-union, and failed prior arthroplasty/infection for both revision TKA and revision THA; (7) preoperative moderate to severe pain and function: assessed by similar questions as detailed above (under outcomes of interest) preoperatively (analyses for pain outcomes were adjusted for preoperative pain and function outcomes (activity limitation) for preoperative function); (8) implant fixation: cemented, hybrid or uncemented, only for primary THA and primary TKA; (9) depression: presence or absence of *International Classification of Diseases*, ninth revision, common modification (ICD-9-CM) codes for depression preoperatively; (10) anxiety: presence or absence of ICD-9-CM code for anxiety preoperatively; and (11) distance from the medical center: <100, 100 to 500 and >500 miles/overseas: distance from the medical center was included, since Mayo Clinic provides TKA/THA to local residents and also a serves as a referral center for patients traveling from afar who may have different disease severity and expectations, and both can impact pain and function outcomes.

### Statistical analyses

Student t and χ^2^ tests were used to compare baseline clinical and demographic characteristics of patients. Responder and non-responder characteristics were compared using logistic regression analyses. Univariate and multivariable-adjusted logistic regression analyses were performed for each outcome at 2 years and 5 years. For these analyses, we used a generalized estimating equations (GEE) approach to adjust for the correlation between observations on the same subject. Eight analyses were performed for each outcome (pain and activity limitation), separately for primary THA, primary TKA, revision THA and revision TKA at the 2-year and 5-year follow-up. To account for potential collinearity, we examined correlation between ASA score and Deyo-Charlson comorbidity index. Since it was <0.4, both were included in the model. The main multivariable-adjusted analyses adjusted for age, gender, BMI, comorbidity, ASA class, operative diagnosis, distance from the medical center and preoperative pain/function in all models; implant fixation was added only to models for primary THA/TKA. Sensitivity analyses were performed for each of these analyses by additionally adjusting for anxiety and depression, since psychological factors have been shown to impact pain and function outcomes after THA and TKA. Another set of sensitivity analyses were performed limiting the primary THA or primary TKA cohorts to those with osteoarthritis as the underlying diagnoses, to examine whether the underlying diagnosis had any major impact on the study findings. We present only the multivariable-adjusted estimates for the main models for the ease of understanding. Odds ratios and 95% confidence intervals are presented. A *P* value <0.05 was considered significant.

## Results

### Patient characteristics

For the 2-year follow-up (n = 3,823), the mean age of the primary THA cohort was 65 years, 48% were men, 31% were ≤60 years and 36% had a BMI of 30 kg/m^2^ or higher. Similarly, for the primary TKA cohort with 2-year follow-up (n = 4,701) the mean age was 68 years, 44% were men, 18% were ≤60 years and 52% had a BMI of 30 kg/m^2^ or higher (Table 1).

**Table 1:** Clinical and demographic characteristics of patients with primary total hip or knee arthroplasty (THA/TKA)

**Characteristic**	**Primary THA**		**Primary TKA**	
	**2 years**	**5 years**	**2 years**	**5 years**
	**(n = 3,823)**	**(n = 2,374)**	**(n = 4,701)**	**(n = 2,935)**
Mean age ± SD	64.8 ± 13.2	64.3 ± 12.8	68.4 ± 9.5	68.5 ± 9.1
Men/women (%)	48%/52%	48%/52%	44%/56%	45%/55%
Age groups, n (%):				
≤60 years	31%	31%	18%	17%
>60 to 70 years	31%	33%	36%	39%
>70 to 80 years	29%	28%	38%	38%
>80 years	8%	6%	7%	6%
Body mass index, kg/m^2^				
≤25 (normal)	25%	24%	13%	13%
>25 to 29.9 (overweight)	39%	40%	35%	36%
30 to 34.9 (mildly obese)	23%	23%	30%	30%
35 to 39.9 (obese)	8%	8%	14%	14%
≥40 (morbidly obese)	4%	4%	8%	7%
ASA score:				
Class I to II	63%	65%	59%	60%
Class III to IV	36%	35%	41%	40%
Implant fixation:				
Cemented	10%	12%	98%	100%
Hybrid	55%	60%	0%	0%
Uncemented	35%	28%	0%	0%
Underlying diagnosis				
Inflammatory arthritis	2%	3%	3%	4%
Osteoarthritis	88%	86%	96%	93%
Avascular necrosis of bone	7%	7%	-	-
Other^a^	3%	4%	2%	3%

All numbers were rounded to the nearest digit, therefore totals may not exactly add up to 100%.

Other^a^ category includes the following: for primary THA: hip dysplasia, Legg-Perthe’s disease, slipped capital femoral epiphyses, failed previous osteotomy, failed previous arthrodeses, failed previous internal fixation, congenital dislocation of hip, pigmented villonodular synovitis, hemochromatosis, synovial chondromatosis, and so on; for primary TKA: genu varum, genu valgum, hemophilia, Paget’s disease, failed previous disease including arthrodesis, failed previous osteotomy, failed previous patellectomy, Chacot arthropathy, chondromalacia, pigmented villonodular synovitis, and so on.

ASA, American Society of Anesthesiologists.

The 2-year revision THA cohort (n = 1,218) had a mean age of 66 years, 46% were men, 31% were ≤60 years and 30% had BMI of 30 kg/m^2^ or higher (Table 2). The 2-year revision TKA cohort (n = 725) had mean age of 69 years, 51% were men, 20% were ≤60 years and 50% had BMI of 30 kg/m^2^ or higher.

**Table 2:** Clinical and demographic characteristics of patients with revision total hip arthroplasty (THA) or revision total knee arthroplasty (TKA)

**Characteristic**	**Revision THA**		**Revision TKA**	
	**2 years**	**5 years**	**2 years**	**5 years**
	**(n = 1,218)**	**(n = 727)**	**(n = 725)**	**(n = 393)**
Mean age ± SD	65.8 ± 12.9	64.6 ± 13.3	68.7 ± 9.9	67.8 ± 10.2
Men/women (%)	46%/54%	45%/55%	51%/49%	53%/47%
Age groups, n (%):				
≤60 years	31%	33%	20%	22%
>60 to 70 years	26%	27%	32%	29%
>70 to 80 years	34%	34%	40%	41%
>80 years	9%	6%	8%	7%
Body mass index, kg/m^2^:				
≤25 (normal)	29%	28%	12%	11%
>25 to 29.9 (overweight)	39%	41%	38%	38%
30 to 34.9 (mildly obese)	21%	21%	28%	29%
35 to 39.9 (obese)	6%	6%	15%	14%
≥40 (morbidly obese)	3%	3%	7%	7%
ASA score:				
Class I to II	56%	63%	55%	59%
Class III to IV	44%	37%	45%	41%
Underlying diagnosis:				
Loosening/wear or osteolysis	73%	75%	62%	64%
Dislocation, bone or prosthesis Fracture, instability, non-union	17%	15%	25%	24%
Failed prior arthroplasty with components removed or infection	11%	11%	13%	11%

ASA, American Society of Anesthesiologists.

All numbers were rounded to the nearest digit, therefore totals may not exactly add up to 100%.

### Prevalence of ipsilateral knee/hip involvement

Ipsilateral knee involvement was reported in 11% patients at 2 years and 16% patients at 5 years after primary THA. The respective proportions after revision THA were 18% at 2 years and 17% at 5 years. Ipsilateral hip involvement was reported by 12% at 2 years and 13% at 5 years after primary TKA and by 16% each at 2 years and 5 years after revision TKA.

### Ipsilateral knee/hip involvement and multivariable-adjusted outcomes after primary THA/TKA

Patients with ipsilateral knee involvement had 130% higher adjusted odds of moderate to severe index THA pain at 2 years and 80% higher odds at 5 years after primary THA, both statistically significant (Table 3). Ipsilateral knee involvement increased the odds of moderate to severe functional limitation by 210% at 2 years and 260% at 5 years after primary THA (Table 3).

**Table 3:** **Multivariable-adjusted**^**a **^**association of ipsilateral knee/hip involvement with outcomes after primary total hip or knee arthroplasty (THA/TKA)**

	**THA**				**TKA**			
	**2 years**		**5 years**		**2 years**		**5 years**	
	**Odds ratio (95% CI)**	***P *value**	**Odds ratio (95% CI)**	***P *value**	**Odds ratio (95% CI)**	***P *value**	**Odds ratio (95% CI)**	***P *value**
Primary:								
Moderate severe pain (ref, none)	2.3 (1.5 to 3.6)	<0.001	1.8 (1.1 to 2.7)	<0.01	3.3 (2.3 to 4.7)	<0.001	1.8 (1.1 to 2.7)	<0.01
Moderate severe activity limitation (ref, none)	3.1 (2.3 to 4.3)	<0.001	3.6 (2.6 to 5.0)	<0.001	3.6 (2.6 to 4.9)	<0.001	2.2 (1.6 to 3.2)	<0.001
Revision:								
Moderate severe pain (ref, none)	1.9 (1.1 to 3.1)	0.01	2.2 (1.2 to 4.0)	<0.01	2.0 (1.1 to 3.8)	0.02	NE^b^	-
Moderate severe activity limitation (ref, none)	3.6 (2.2 to 5.8)	<0.001	8.8 (4.3 to 17.6)	<0.001	2.9 (1.4 to 5.8)	0.003	2.3 (1.0 to 5.0)	0.043

Adjusted^a^ for age, gender, body mass index, comorbidity, American Society of Anesthesiologists (ASA) class, operative diagnosis, distance from the medical center, preoperative pain (for pain outcomes) and preoperative function (for function outcomes); additionally adjusted for implant fixation (cemented or not) in primary THA.

NE^b^, not estimable (too few cases with this outcome).

Patients with ipsilateral hip involvement had significantly higher odds of moderate to severe index TKA pain by 230% at 2 years and 80% at 5 years after primary TKA (Table 3). Patients with ipsilateral hip involvement had 260% higher odds of moderate to severe functional limitation at 2 years and 120% higher odds at 5 years after primary TKA.

### Ipsilateral knee/hip involvement and multivariable-adjusted outcomes after revision THA/TKA

Patients with ipsilateral knee involvement had 90% higher odds of moderate to severe index THA pain at 2 years and 120% higher odds at 5 years after revision THA (Table 3). Ipsilateral knee involvement increased the odds of moderate to severe functional limitation by 260% at 2 years and 780% at 5 years after revision THA.

Ipsilateral hip involvement was associated with 100% higher odds of moderate to severe index TKA pain 2 years after revision TKA (Table 3). Ipsilateral hip involvement increased the odds of moderate to severe functional limitation by 190% at 2 years and 130% at 5 years after revision TKA.

### Sensitivity analyses

Sensitivity analyses that limited the cohort to only patients with underlying diagnosis of osteoarthritis showed minimal change in odds ratios and no change in the level of significance (Additional file 1). Sensitivity analyses that additionally adjusted the above multivariable analyses for anxiety and depression found that odds ratios either did not change at all or changed minimally, with no change in the level of significance (data not shown). Sensitivity analyses that excluded patients with contralateral joint involvement showed minimal change in odds ratio and no change in the level of significance (data not shown).

### Non-response bias

Response rates at 2 years and 5 years for each cohort were as follows: primary THA, 62% and 57%; primary TKA, 65% and 57%; revision THA, 58% and 48%; and revision TKA, 57% and 48%.

We compared the characteristics of responders and non-responders. Compared to non-responders, patients who responded to the 2-year post-primary THA survey were more likely to be older, have lower ASA class I or II, lower Deyo-Charlson index or live <100 miles from the medical center. At 5-year follow-up, compared to non-responders, responders were more likely to be older, have a higher BMI, lower ASA class I or II, or live <100 miles from the medical center.

Compared to non-responders, survey responders at 2 years after revision THA were more likely to be have higher BMI, lower ASA class I or II, or an operative diagnosis of loosening/wear/osteolysis. At 5 years, compared to non-responders, responders were more likely to have lower Deyo-Charlson index, lower ASA class I or II, or an operative diagnosis of loosening/wear/osteolysis.

For primary TKA 2-year and 5-year follow-up, men and those with osteoarthritis as the underlying diagnosis were slightly more likely to respond to the survey and older age was associated with significantly greater odds of survey response. Higher ASA class of III or IV and higher Deyo-Charlson comorbidity index score were associated with slightly lower and distance of >500 miles from the Mayo Clinic with much lower odds of response. Similar patterns were noted in responder and non-responder patients who underwent revision TKA.

## Discussion

This is the first study to report that ipsilateral lower extremity joint involvement is associated with significantly higher odds of moderate to severe index arthroplasty pain and moderate to severe functional limitation at 2 years and 5 years after index THA and TKA, both in primary and revision cases. The findings were robust and effect sizes were consistent across the type of arthroplasty (hip or knee), primary and revision arthroplasty, and the two follow-up time-points. Sensitivity analyses that adjusted for additional covariates (anxiety and depression), or restricted to the patients with osteoarthritis confirmed the findings. Several findings in this study deserve further discussion.

A key finding of our study was that concomitant ipsilateral knee/hip involvement increased the risk of moderate to severe index arthroplasty joint pain, unequivocally an undesired outcome of THA/TKA, at both 2 years and 5 years after primary THA/TKA. In the absence of any previous studies, these data add new knowledge. Potential mechanisms include referred pain from their involved ipsilateral knee/hip to the index THA/TKA, altered biomechanics and more weight bearing on the index THA/TKA, and limited ability to do adequate rehabilitation and strengthening due to concomitant ipsilateral joint involvement. A causal relationship cannot be inferred due to the assessment of ipsilateral involvement and pain/function outcomes cross-sectionally.

Several underlying conditions can lead to the involvement of the ipsilateral knee/hip, such as [1] osteoarthritis or other arthritis in multiple joint in patients with primary THA and TKA; [2] a failing primary or revision arthroplasty in the ipsilateral joint; and [3] diseases of periarticular structures, such as bursitis or tendinitis, that lead to articular and periarticular symptoms. Future studies should assess whether the treatment of ipsilateral joint involvement leads to improvement in outcomes related to index TKA/THA joint. Whether treatment of activity limitation related to ipsilateral knee/hip with physical therapy, surgical (that is, arthroplasty) or other modalities can improve index THA/TKA outcomes remains to be seen.

Ipsilateral knee/hip involvement had an even stronger relationship with functional limitation following primary THA/TKA than its association with moderate to severe pain. This finding is not unexpected. The higher the number of involved joints in the lower extremity, the more likely it is that a patient will have moderate to severe activity limitation, since these limitations are specific to lower extremity joint and muscle function. To the best of our knowledge this is the first study to assess the impact of ipsilateral joint involvement on TKA/THA pain and function outcomes, using robust analyses. This finding adds to the recent findings that concomitant contralateral knee pain is associated with poorer post knee replacement function, both when present preoperatively [10] and postoperatively [9]. These observations indicate that it is important to pay attention to other joint involvement in patients with suboptimal outcome after THA/TKA. This study does not answer a critical question whether the presence of ipsilateral knee/hip involvement leads to the suboptimal outcome in index THA/TKA, which needs to be examined in future studies. Whether properly addressing concomitant contralateral or ipsilateral joint involvement might improve the pain and functional outcome of the operated joint remains to be seen. Thus, these findings have implications for improving care and potentially outcomes of patients undergoing THA/TKA.

The association of ipsilateral knee/hip involvement with poor pain and function outcomes noted in primary THA/TKA was also noted in patients who underwent revision THA/TKA. In particular, the strength of association after revision THA/TKA was similar to that noted in patients undergoing primary THA/TKA. In addition, we noticed little or no attenuation of the association noted at 2-year follow-up at the longer 5-year follow-up. These findings support the robustness of this association.

The study findings must be interpreted considering study strengths and limitations. Study strengths include a large cohort, prospective standardized data collection by dedicated clinical registry staff, adjustment for important covariates and confounders, and the robustness of findings across several sensitivity analyses. Our study also has several limitations, however. Non-response and referral bias may limit our ability to generalize these findings to other populations. However, patient demographics are similar to previously published studies of primary and revision THA and TKA. The response rate at 2 years is similar to the mean response rate of 60%, typical for large surveys of this size [22]. The response rate at 5 years at 48% is low, and therefore these findings should be interpreted with caution. In general, non-responders were more likely to be younger, obese, higher comorbidity, higher ASA class and live at a greater distance from the Mayo Clinic, characteristics associated with poorer pain and/or functional outcomes after THA/TKA. However, it is unlikely that the association of ipsilateral joint involvement with index THA/TKA pain and function outcomes differed by these characteristics, in absence of any such published data. Therefore, the direction of impact of non-response bias on our findings is unclear.

The joint registry does not provide detailed data on disease pathology in all other joints, and may miss interval arthroplasty in the ipsilateral joint if performed at another institution and this was not reported by the patient in their mailed survey response or telephone interview. Therefore, we are unable to comment on the underlying disease/pathophysiology responsible for ipsilateral joint involvement. Another limitation is that diagnoses of comorbidities were based on the presence of respective ICD-9 codes, making underdiagnosis and misclassification bias likely. However, misclassification would bias our findings towards null; therefore our estimates are conservative. We made an ‘*a priori*’ decision to combine moderate and severe categories based on our clinical judgment of what would be considered suboptimal by operating surgeons, but also to have enough events to analyze predictors of poor outcomes. Despite our efforts to include several important variables, residual confounding is possible.

## Conclusions

In summary, we found that ipsilateral knee/hip involvement was a significant predictor of moderate-severe pain and moderate-severe activity limitation after THA/TKA. These findings have important implications for patients and surgeons. In patients with poor pain and function outcomes, addressing the ipsilateral joint involvement may improve the outcome of the operated joint. Future studies should examine the effect of prevention and treatment of ipsilateral pain on outcome of the index joint arthroplasty.

## Competing interests

There are no financial conflicts related directly to this study. JAS has received research and travel grants from Takeda and Savient and consultant fees from URL pharmaceuticals, Savient, Takeda, Ardea, Regeneron, Allergan and Novartis. DGL has received royalties/speaker fees from Zimmer, has been a paid consultant to Zimmer and has received institutional research funds from DePuy, Stryker and Zimmer. The views expressed in this article are those of the authors and do not necessarily reflect the position or policy of the Department of Veterans Affairs or the United States government.

## Authors’ contributions

Study design and protocol: JAS. Review of study design: JAS, DGL. Data analyses: JAS. Review of analyses and results: JAS, DGL. Manuscript draft: JAS. Manuscript revision: JAS, DGL. Submission: JAS. All authors read and approved the final manuscript.

## Pre-publication history

The pre-publication history for this paper can be accessed here:

http://www.biomedcentral.com/1741-7015/11/144/prepub

## Supplementary Material

Additional file 1 Sensitivity analyses for primary THA and primary TKA cohorts limiting only to patients with osteoarthritis (OA). Click here for file
