# Supplementary material for: Ipsilateral lower extremity joint involvement increases the risk of poor pain and function outcomes after hip or knee arthroplasty
Source: BMC Med. 2013 Jun 5;11:144. doi: 10.1186/1741-7015-11-144 (PMC3681648; doi:10.1186/1741-7015-11-144)
Supplement: Additional file 1 — Sensitivity analyses for primary THA and primary TKA cohorts limiting only to patients with osteoarthritis (OA). [file 1741-7015-11-144-S1.docx]

**ADDITIONAL FILE**

**Additional file 1. Sensitivity analyses for primary THA and primary TKA cohorts limiting only to patients with osteoarthritis (OA).**

|  | **Primary THA** | | | | **Primary TKA** | | | |
| --- | --- | --- | --- | --- | --- | --- | --- | --- |
|  | **2-year** | | **5-year** | | **2-year** | | **5-year** | |
|  | **Odds Ratio (95% CI)** | **p-value** | **Odds Ratio (95% CI)** | **p-value** | **Odds Ratio (95% CI)** | **p-value** | **Odds Ratio (95% CI)** | **p-value** |
| **Moderate severe pain (Ref,** None) | 2.2 (1.4, 3.1) | 0.001 | 1.9 (1.1, 3.0) | 0.008 | 3.4 (2.4, 4.9) | <0.001 | 1.8 (1.1, 2.8) | 0.012 |
| **Moderate severe functional limitation (Ref,** None) | 3.1 (2.2, 4.2) | <0.001 | 3.7 (2.6, 5.2) | <0.001 | 3.6 (2.6, 4.9) | <0.001 | 2.3 (1.6, 3.2) | <0.001 |
